# Individual's Experience of Living With Roux‐en‐Y Gastric Bypass Surgery: An Interpretative Phenomenological Analysis

**DOI:** 10.1111/jan.16890

**Published:** 2025-03-25

**Authors:** Nathan Faulkner, Andreas Vassiliou, Joanne Lusher

**Affiliations:** ^1^ Regent's University London London UK; ^2^ Private Practice Nicosia Cyprus

**Keywords:** bariatric surgery, gastric by‐pass, nurse roles, phenomenology, qualitative, Roux‐en‐Y, weight loss

## Abstract

**Aim:**

To explore the lived experience following Roux‐en‐Y gastric bypass surgery of eight men and women in the South of England who had undergone surgery a minimum of 12 months prior.

**Design:**

This phenomenologically based qualitative study utilised Interpretative Phenomenological Analysis (IPA) as a framework for the analysis and exploration of participants' lived experiences.

**Method:**

Semi‐structured individual interviews were conducted with eight men and women in the South of England in 2017.

**Results:**

Interpretative Phenomenological Analysis revealed four superordinate themes: Managing change and uncertainty; The affective experience of change; The post‐operative body within its relational context; and The presence and absence of appropriate support.

**Conclusion:**

Participants described the complex nature of the post‐operative experience and highlighted the deeply personal nature of the adjustment required following surgery. The process of change and adjustment does not represent a smooth transition from pre‐ to post‐operative life, and the experience of weight loss is intertwined with relationships that require patients to renegotiate the ways in which they understand themselves within social encounters.

**Implications and Contribution:**

The clinical significance of this study lies in its support for the contribution that an existential phenomenological approach can offer in supporting individuals who choose to have bariatric surgery through its acknowledgment of the body as a site of experience which is situated within a person's wider social, cultural and historical world. These findings contribute an in‐depth appreciation of the biopsychosocial experiences of individuals following Roux‐en‐Y gastric bypass surgery that can be applied in nursing practice to better inform the development of appropriate ways in which to support the overall wellbeing of individuals who made the decision to undergo bariatric surgery.

**Patient and Public Contribution:**

Limited patient involvement was incorporated, focusing on feedback on the interview process.

**Summary**

- Why is this research needed?
  ○ Roux‐en‐Y gastric bypass surgery is the second most common bariatric procedure internationally.
  ○ Importance of qualitative research for exploring individuals' lived experiences following bariatric surgery.
  ○ Interpretative phenomenological analysis offers potential for exploring participants' view of the world and perspective of the phenomenon under study.
- How do the findings from this research contribute to the field?
  ○ Provides important insight into longer‐term experiences following Roux‐en‐Y gastric bypass surgery.
  ○ Supports the use of an existential phenomenological approach for supporting individuals undergoing bariatric surgery through its acknowledgment of the body as a site of experience.
  ○ Highlight the deeply personal nature of changes and struggles experienced after undergoing gastric by‐pass surgery and the ways in which these are intertwined with a person's social, cultural and personal history.
- How can the findings be used to inform policy/practice?
  ○ Supports previous research suggesting that a patient's perceived suitability for surgery pre‐operatively might bear little relation to their post‐operative experience of change.
  ○ Emphasises the importance of post‐operative support addressing the individuals' experience of adjustment and a need the move away from focusing on weight loss as a marker of success to a more holistic view of the person's overall wellbeing post‐operatively.
  ○ Highlights the important role nurses have in supporting the psychological wellbeing of bariatric surgery patients by ensuring a biopsychosocial approach to post‐operative care is implemented.

## 1 Introduction

Recent figures from the International Federation for the Surgery of Obesity and Metabolic Disorders (IFSO) report that internationally, approximately 159,543 Roux‐en‐Y gastric bypass (RYGB) procedures were performed in 2021, making this procedure second only to sleeve gastrectomy (Angrisani et al. 2024). Despite Roux‐en‐Y gastric bypass (RYGB) surgery typically resulting in significant and rapid weight loss and reductions in medical risk factors, these physical changes are accompanied by equally important psychosocial changes. These adjustments include the need to adapt eating behaviours alongside alterations in perceived body image, psychological and emotional wellbeing and interactions with others and social relationships (de Lourdes et al. 2021; Chan et al. 2020; Coulman et al. 2020), of which far less is known. Recognising the limitations of standardised measures to capture the complex nature of the impact of gastric bypass on overall quality of life, qualitative research provides a shift away from predicting outcomes using statistical data to the use of in‐depth language that offers potential by exploring the nuanced ways in which individuals experience being overweight (Sillén and Andersson 2017; Imhagen et al. 2023) and how they navigate life post‐operatively (Chan et al. 2020). Based on recent findings (Coulman et al. 2017; MacAskill et al. 2023), qualitative research offers valuable perspectives on the impact of RYGB surgery on the life of the individual.

In positioning this study, we draw on the qualitative findings of a study by Ogden et al. (2006) who sought to understand patients' experiences of undergoing surgery and the impact on quality of life and eating. These authors described how participants recounted the ways surgery had impacted their eating behaviour and relationship with food with some describing a mind–body split where ‘the stomach is now controlling the mind’ (p. 256) by preventing overeating through signalling fullness, whereas for others, whilst they also recognised their body exercising restriction, they had been able to internalise this sense of control themselves. Further, Natvik et al. (2013) interviewed participants several years following surgery. Natvik and colleagues described their findings as highlighting ways in which participants felt ‘totally changed, yet still the same’ capturing not only a sense of ambivalence but also how the restriction immediately after surgery represented both a long‐awaited improvement yet at the same time an abrupt transition which left them struggling to adjust to their altered body. Despite participants feeling relieved at their weight loss and smaller bodies, changes in body shape and loose skin created an uncertainty regarding the extent their smaller body conformed to *normal* bodies within society.

The social and emotional nature of eating has been explored (Goodspeed‐Grant 2008; Delormier et al. 2009; Oskis 2022) and might account for the ambivalence participants described between surrendering themselves entirely to the surgery in the pursuit of long‐term weight loss and the refusal to relinquish themselves entirely to the care of healthcare services. The sense of ‘being at the mercy of one's body’ (Natvik et al. 2014, 1706) was found to confront participants with challenges in finding time for self‐care alongside the day‐to‐day necessities of their lives such as work and relationships, an experience the authors describe as like ‘being sentenced to inescapable awareness of their habits, sensations, emotions, and thoughts’ (p. 8). This ambivalence between participants' desire to maintain weight‐loss and urges to eat signified a split within their relationship with eating in which they noticed being able to still engage in previously problematic behaviours such as binge eating. Whilst the return of ‘problematic’ eating behaviours resulted in confusion for some, others realised how the increased awareness of their bodies had resulted in them re‐embodying their new habits. By adopting an existential phenomenological lens within their analysis, Natvik et al. (2013, 2014) capture the ambiguity which characterises the long‐term experience of life following bariatric surgery and the tensions individuals experience between relinquishing and maintaining control.

As can be seen from the studies presented so far, far from being discrete behavioural factors that can be changed in isolation, changes to eating practices following surgery are embedded within a person's wider life. Furthermore, many individuals who undergo surgery experience severe adverse consequences in relation to eating because of side effects such as those experienced during episodes of dumping; a cluster of symptoms including irregular heartbeat, diarrhoea, drop in blood pressure and vomiting resulting from food arriving rapidly at the small intestine triggering a series of physiological responses (Masclee and Masclee 2023). Exploring the lived experience of dumping, Groven et al. (2012) challenged previous studies that suggested an increased sense of control (Ogden et al. 2006) and feeling of disburdening (Engström and Forsberg 2011) and instead described an ongoing sense of ambivalence where the experience of dumping was experienced as an unpleasant bodily experience whilst simultaneously being described as a ‘warning system’ (p. 45).

Engström and Forsberg (2011) described how dumping manifested in both an awareness of changes to participants ‘inner body’ and as an inter‐relational phenomenon which participants had to learn to manage in different contexts. Participants described unpredictability as one of the greatest challenges after surgery and how in attempts to avoid dumping they would spend a lot of time and energy exploring what food and drinks their bodies could tolerate. Conceptualising the way in which dumping represents both an internal and inter‐personal phenomenon, Groven et al. expand upon Merleau‐Ponty's perspective of the ‘lived body’ using Leder's notions of the ‘inner body’ and ‘surface body’ which acknowledge the unity of the internal and external body in defining its position in the world through the ‘ceaseless stream of kinesthesia, cutaneous and visceral sensations’ (1990, p.23). Adopting this perspective, the authors emphasise the ways in which the experience of dumping emanating from physiological changes represent far more than unpleasant side‐effects and are inextricably intertwined with all areas of the person's Being.

From this brief introduction of phenomenological studies exploring individuals' post‐operative experience, far from being a procedure which merely reduces the size of the stomach and the amount a person can eat, the physical changes following surgery confront the person with the need to adjust and renegotiate their sense of their body in the world. Although studies have tended to focus on the specific aspects of change it is evident that none of these areas are experienced within a vacuum following surgery but instead are intertwined, both with each other and within the individual's history, current life situation and how they project themselves into the future. In attempting to address some of the issues raised within the current literature, this study explored the lived experience of men and women who underwent RYGB surgery at least 12 months prior to taking part in the study. Given the perceived issues with support following surgery reported in previous research, attention was given to the ways in which participants utilised support post‐operatively.

## 2 Design

This study used semi‐structured interviews with individuals who had undergone RYGB surgery. Consistent with IPA's idiographic commitment, the sample recruited within this study is consistent with qualitative research that has a focus on honouring the ‘rich and unique stories’ presented by participants (Wertz 2005; Smith et al. 2009). Interviews were analysed using the stages set out by Smith et al. (2009) for use when conducting an IPA. Given the complex nature of change individuals experience following bariatric surgery, a phenomenological approach offered the opportunity to explore the participant's view of the world and to adopt, as far as is possible, an ‘insider's perspective’ of the phenomenon under study (Smith 1996, 264). With its aim to grasp the ‘texture and qualities of an experience as it is lived by an experiencing subject’ (Eatough and Smith 2008, 3), along with its origins within healthcare research, IPA represented an appropriate methodology for exploring the gap between an object and the persons perception of it, which can be seen most evidently when considering real bodies and the persons accounts of physical processes (Smith 1996).

### 2.1 Recruitment

Information for participants was circulated to the lead dietician within the bariatric service. Working in collaboration with the dietician allowed for a more purposive approach to sampling as all patients accessing the service who met the inclusion criteria of having undergone RYGB surgery at least 12 months prior were informed of the research during routine follow‐up appointments and provided with information including contact details. During the initial communications, confidentiality was explained, and participants were reassured that the bariatric service would not have access to any information provided by them during their participation, and written consent was obtained prior to each interview.

### 2.2 Participants

Eight participants were recruited from a specialist bariatric surgery unit, five female and three male, who had undergone RYGB surgery at least 12 months prior to taking part in the research interview. This group was chosen as following surgery it is commonly acknowledged that the weight loss resulting directly from the surgical intervention usually begins to decline around 12 months post‐operatively and therefore represents a transitional stage in which individuals are required to maintain the lifestyle changes necessary for continued weight loss and weight loss maintenance (Bocchieri et al. 2002; Lynch 2016; Magro et al. 2008; Wimmelmann et al. 2014). Previous research has tended to focus on the initial 12 months following surgery (Coulman et al. 2017); therefore, this study sought to engage with a phenomenological exploration of individual's experience living with RYGB surgery beyond this phase and the ways in which participants managed this transition period. By adopting this focus, this research ensured the participants' lives represented a *revelatory relationship* with the phenomena being studied (Wertz 2005).

Participants, described in Table 1, were recruited through the UK National Health Service (NHS). Given the need for interview transcripts to be thoroughly transcribed, it was necessary for participants to be English‐speaking. Due to the combined restriction and malabsorption experienced by individuals following the RYGB procedure and the potential consequences of non‐compliance, only individuals who had undergone this type of bariatric procedure were recruited.

**TABLE 1:** Participant demographics.

Participant pseudonym	Gender	Age	Ethnic origin	Relationship status	Duration of relationship	Months since surgery
Robert	Male	63	White British	Married	18 years	36 months
Stephen	Male	55	White British	Living with partner	19 years	19 months
Tony	Male	62	White British	Divorced	32 years	24 months
Jenny	Female	47	White British	Separated	10 years	24 months
Joyce	Female	57	White British	Divorced	6 years	15 months
Bronwyn	Female	37	White British	Married	14 years	24 months
Helen	Female	50	White British	Civil partnership	Incomplete	36 months
Sophie	Female	42	White British	Single	42 years	21 months

### 2.3 Data Collection

Interviews were guided by a semi‐structured schedule. Honouring the idiographic nature of IPA, the schedule reflected a basis for conversation as opposed to a prescriptive or definitive list of topics to be covered in the interview, allowing for participants to talk about what was most relevant for them in their post‐operative experience (Larkin et al. 2006). Consistent with the approach proposed by Smith et al. (2009) the intention was to allow participants to take the lead during the conversation. Individual interviews were completed by the first author and were recorded using a Dictaphone. Where participants commented upon a potentially poignant experience, this was noted to refer to at an appropriate time, and field notes were completed following each interview to ensure notable features of the interview process were captured. The first author is a registered psychotherapist and psychologist with experience of carrying out qualitative research.

Interviews were opened with a broad enquiry about experiences of weight across a lifetime. Opening with a broad enquiry such as this was intentional as, from a phenomenological perspective, time is subjective and therefore offered grounds upon which participants' decision for surgery might exist. Following the participants lead, follow‐up questions and prompts were used to further explore responses and experiences. Interviews took place within participants' homes over a two‐month period in 2017.

### 2.4 Analysis

Interviews were audio recorded and transcribed by the first author. Whilst time intensive, this process allowed for full immersion into the transcripts, enabling the capture of subtleties in expression, both verbal and non‐verbal, from field notes, as well as recognising the researcher's own reactions to the text. The analysis was a long and deeply analytical one, with a series of steps that were followed and returned to throughout the analytical process.

#### 2.4.1 Step 1

The first author transcribed the interviews to allow them to pay meticulous attention to accurately capturing ‘indications of pauses, mis‐hearings, apparent mistakes, and even speech dynamics where these are in any way remarkable’ (Biggerstaff and Thompson 2008, 217). Once transcribed, all transcripts were read twice whilst simultaneously listening to the recordings to pay close attention to vocal intonations, pauses and silences and gain a more thorough understanding of the participants account (Wertz 2005). Consistent with the idiographic commitment of IPA each transcript was read and coded individually before progressing to the next.

#### 2.4.2 Step 2

Although some noting had taken place during Stage 1, this stage reflected a more textual analysis. Smith et al. (2009) suggest three analytic lenses to guide commentary on the text during this notetaking stage: *descriptive* comments on content, *linguistic* exploring the participants use of language, and *conceptual* reflecting the hermeneutic of questioning where the focus may move away from the participants' words and become more interpretative. The sections of text that comments related to varied in length between a few words and an entire paragraph.

#### 2.4.3 Step 3

Using the detailed comments, thoughts and reflections developed in previous stages, this stage represents an attempt to reduce the amount of data whilst maintaining complexity, in terms of mapping the interrelationships, connections and patterns between exploratory notes (Smith et al. 2009). To ensure transparency within the audit trail, the first author maintained a record of the line number and quote that supported each of the emergent themes.

#### 2.4.4 Step 4

Once a set of emergent themes had been identified for each participant, the first author considered how these fitted together. Smith et al. (2009) suggest three ways of looking for connections and divergences within and between interviews: *abstraction*, which refers to placing like with like; *subsumption*, wherein an emergent theme acquires superordinate status within the grouping process; and *polarisation*, which seeks to identify oppositional relationships between themes. Once a representation of the emergent themes was developed, the first and second authors reflected upon the emergent themes together. This process represented a function of the hermeneutic circle by allowing the first author space to momentarily step outside of their immersion in the data to consider the participants' account as a whole, which subsequently helped in identifying the parts that felt most prominent.

#### 2.4.5 Step 5

This process was repeated for each participant. Consistent with the idiographic foundation of IPA, the process of reading and re‐reading while simultaneously listening to the interview was useful in engaging with each subsequent interview and reflecting on the researcher's own experience of being with each participant.

#### 2.4.6 Step 6

Concerned with looking for patterns across participants. After all interviews had been analysed, the first author looked across groupings for each participant, looking for patterns and divergence.

#### 2.4.7 Rigour

To ensure trustworthiness within qualitative research, Yardley's (2000) principle of rigour was adhered to. Yardley (2000) refers to rigour within the analytic process as ‘the use of a prolonged contemplative and empathic exploration of the topic together with sophisticated theorizing’ (p. 222). Within the study, this principle was achieved by the first author's close engagement with the participants' narratives, a process further supported by working closely with the second author who was experienced in using IPA. Additionally, this research adopted the guidelines outlined by Elliott et al. (1999) for reporting qualitative research.

### 2.5 Ethical Considerations

Ethical approval was received from both Metanoia Institute (MREC Ref: 14/14–15) on 30th July 2015 and NHS West of Scotland Research ethics Service (REC Ref: 182083) on 27th November 2015. Acknowledging the existence of powerful stigmatising discourses surrounding obesity (Puhl and Brownell 2003; Hooper et al. 2012; Dimitrov Ulian et al. 2023) there existed the potential for participants to experience the research as either pathologising, stigmatising or for feelings of guilt or shame regarding their decision to undergo surgery (Faulkner and Lusher 2024a). Confidentiality and participants right to withdraw without any impact on their ongoing care within the bariatric service were made explicit.

## 3 Results

Listed in Table 2 are the four superordinate themes and 13 subthemes identified within the participants accounts of living with their RYGB: Managing change and uncertainty; The affective experience of change; The post‐operative body within its relational context; and The presence and absence of appropriate support.

**TABLE 2:** Table of superordinate and emergent themes.

Superordinate themes and related subthemes	Interviews containing this theme							
	1	2	3	4	5	6	7	8
1. Managing change and uncertainty								
1.1 Renegotiating the body post‐operatively		•	•	•	•	•		•
1.2 Experience of adjusting to imposed restriction	•		•	•	•	•	•	•
1.3 Redefining relationship to food	•		•	•	•	•	•	
1.4 Putting surgery into perspective		•	•	•	•	•	•	•
2. The affective experience of the body post‐operatively								
2.1 Disillusionment with hoped for transformation	•	•	•	•	•	•		•
2.2 Living in a state of limbo	•		•	•	•	•	•	•
2.3 Enduring sense of self as overweight	•			•	•	•	•	
3. The post‐operative body within its relational context								
3.1 Feeling of becoming normal		•		•	•	•	•	
3.2 Managing other's reactions to transformed body		•		•	•	•	•	•
3.3 The solitary burden of the post‐operative body	•		•	•	•	•		•
4. The presence and absence of appropriate support								
4.1 The absence of appropriate professional support	•	•	•		•	•	•	•
4.2 The privileging of the body in post‐operative support	•	•				•	•	•
4.3 The importance of peer support	•	•		•	•	•		•

### 3.1 Managing Change and Uncertainty

The essence of this theme relates to the ways in which participants notice their bodies having changed following surgery and the ways in which they were now required to respond to these changes at cognitive, behavioural and emotional levels. Whilst a sense of loss around food preferences and eating practices characterised some participants' experiences, others also identified ways in which their lives had been enriched through the process of adapting. Whilst participants identified changes that they sometimes found difficult to negotiate, the sense of uncertainty experienced in the present was set against a belief in a certain future that would have awaited them had they not undergone bariatric surgery.

#### 3.1.1 Renegotiating the Body Post‐Operatively

Participants acknowledged ways in which their bodies were no longer the same and now required attention and consideration in ways that they had not previously required, particularly with regards to their bodies' capacity to accept food. For some these changes represented a challenge whereas for others they resulted in a sense of frustration and realisation of the responsibility that undergoing gastric by‐pass surgery involves. Participants' statements within this theme capture a relational quality where they find themselves having to negotiate not only what their bodies will accept but also the demands it makes on their time and attention.

The dietician did turn round and say, she said you seem to be trying things whereas some people just sit there and go ‘Oh I don't know what to do’. You know I will try. You know, it's the same problem if you've got a problem with a car, you've got to try certain things until you get it right you know and that's what I try to do. [Stephen]

#### 3.1.2 Experience of Adjusting to Imposed Restrictions

The second subtheme captures participants' responses to the physical change that appeared to be experienced as *imposed* on their bodies by the surgical procedure. The use of the verb *adjusting* expresses the idiographic nature of these responses in the form of emotional, cognitive and practical reactions. What is interesting in this theme is the way in which most participants refer, either explicitly or implicitly, to a desire to revert to a relationship with food that existed prior to surgery that they wish to escape from yet also crave.

The first three or four weeks you just had liquid food then you went on to a little dish then you went on to a tea plate and it was good because after that you think ‘I'm full up’ and you couldn't eat no more. But now you sort of think ‘oh I could eat a bit more’ you know ‘I need some more’ but I don't, but you could do. The mind says you could do but it's awkward it is really hard. [Robert]

Robert's struggle comprises an apparent dichotomy between his desire to be restricted and his mind's desire to eat more. The tension he refers to between his desire for containment and his mind's contradictory stance is shared by Bronwyn who, despite recognising the benefit of being controlled by the surgical procedure, describes being *angry* and *battling* against the restriction imposed by the gastric by‐pass.

#### 3.1.3 Redefining Relationship to Food

An associated experience to participants' accounts of loss and their need to adjust was observable in the ways they made sense of their altered eating behaviours. Most notably, participants described how they recognised their relationship with food having changed. For some, the ways in which they described having noticed their relationship with food changing was pragmatic, acknowledging the fact that they were unable to enjoy food in the same ways as before and were looking for ways to minimise this loss.

I can no longer physically enjoy it. What do I do? Do you carry on moaning about that, or do you take a different route, you know ? And that's what I've done. [Tony]

#### 3.1.4 Putting Surgery Into Perspective

The final subtheme captures the ways in which, alongside the uncertainty that comes with changes to their bodies and their relationships to food, there exists a certainty that without surgery their lives would have been much worse. Statements reflect a process of positioning the RYGB within the wider contexts of their lives. Stephen conveys a belief that without it, he would not be here now.

I say it time and time again, without it I think I was on a one‐way ticket you know. [Stephen]

### 3.2 The Affective Experience of the Body Post‐Operatively

Whereas the first theme addressed the ways in which participants noticed responding to bodily changes brought about by surgery on cognitive, behavioural and emotional levels, the second superordinate theme captures descriptions of living in their post‐operative body. At its core, this theme conveys the personal and often very private struggles participants experience, including feelings of disappointment with their post‐operative bodies and uncertainty around the future living with a physical state that feels temporary.

#### 3.2.1 Disillusionment With Hoped for Transformation

Participants described varying degrees of disappointment following surgery and the disparity between what they had hoped for and the situation they now found themselves in. For most, the sense of disillusionment centred around their physical appearance and the ways in which this now affects their sense of their bodies on intrapersonal and interpersonal levels. Robert expressed a considerable amount of disappointment and disgust towards his body throughout his interview describing how all he sees when looking at his reflection is a *‘big bulge’* and how in his mind ‘I'm massive. I'm still big’. For him, his physical appearance featured prominently in his disillusionment with how his body had failed to *adjust* in the way he had hoped for following surgery.

#### 3.2.2 Living in State of Limbo

The use of the word *limbo* within this subtheme was used to convey the sense, evident within participants dialogues, of existing within a transitional state, the outcome of which felt uncertain. Of interest within this theme are the ways in which participants convey their experience of living within a body that feels temporary and not entirely their own, which for some results in an increased vigilance towards their current physical state through fear of it changing and for others a sense of futility that embodied change is not possible.

It's always going to be a fight. I suppose, I, I try and look at it on the side of an addict that a drinker is always going to be an alcoholic until the end. And I'm always going to be a [pause] impulsive food eater. That's never going to change. [Bronwyn]

#### 3.2.3 Enduring Sense of Self as Overweight

Sharing the sense of process like experience captured in the previous subtheme where participants described a sense of being between states, the final subtheme relating to participants' affective experience of their bodies post‐operatively highlights the ways in which many of the participants acknowledged feeling that, despite noticeable physical changes, their perception of their bodies remains unchanged from before surgery. Within all their statements, there appeared to exist an underlying split between mind and body that formed a central part of their experience of relating to the changes that have occurred to their bodies post‐operatively.

### 3.3 The Post‐Operative Body Within Its Relational Context

Although the themes have dealt with participants' experience of their bodies on cognitive, emotional and behavioural levels, including the ways in which they both experience and manage the uncertainty that has arisen following their weight loss, what was evident in the data was the way in which the body exists within a relational world which the third superordinate theme addresses. Within this theme, participants describe how they now evaluate their bodies against social categories of *normality*, how they recognise others relating to their physical transformations, and the ways in which, individually, they all experience feelings of isolation arising from their sense of their physical bodies.

#### 3.3.1 Feeling of Becoming Normal

The first subtheme captures the implicit and explicit ways in which participants evaluate their success against constructions of *normality*. For some, this is experienced through the recognition of their body being able to do everyday and sometimes seemingly mundane things that they were unable to do before having surgery, whereas for others, they notice the way in which, following weight loss, their interactions with others feel distinctly different.

I can remember going to Thorpe Park before the surgery with the children, and I couldn't get on any big rides. I was too big to get on any of them and it was awful. The kids were desperate for me to go on so they made me sit in that chair outside the que to try it […] For everyone I had to get into and for everyone I had to turn round and say I can't fit [laughs]. Which was awful. So, to be able to do stuff like that with the children now is, you know. That was one of my goals. [Jenny]

Jenny's desire for her body to be an accepted size highlights an interesting paradox of the contextual nature of acceptability as she explicitly states that, within intimate settings, her body now feels unacceptable as a result of *not* being normal in society.

#### 3.3.2 Managing Others' Reactions to Transformed Body

Whilst the first subtheme addressed participants cognitive and affective experiences of feeling normal within social and relational contexts, participants also spoke about how they experienced other's reactions to their physical changes. Whilst exercising caution in this observation, within this subtheme there appeared to exist a gender difference in ways in which participants described the nature of other's reactions to their post‐operative bodies and the ways in which they managed these. Whereas female participants described the ways in which they noticed their smaller bodies being related to on intimate and sexual levels, the men, with the exception of those closest to them, were somewhat dismissive of others' opinions on their appearance and instead appeared to base their understanding of the changes they experienced on what their bodies were capable of doing.

A few people turn round and say, ‘ooh you don't want to lose anymore’ and I know why that is but I'm not worried […] it's because I look so thin compared to what I used to. That's why it is. I mean, that's all it is. […] I can understand what they're saying but it doesn't affect me in the slightest. [Stephen]

#### 3.3.3 The Solitary Burden of the Post‐Operative Body

A common theme described ways in which participants feel alone in dealing with their body and how this sense of aloneness now resides as a burden they are required to bear. Robert expressed openly his dissatisfaction with his body following surgery, appearing to focus extensively on discrete parts of his body, such as the *bulge* referred to below, which persisted despite him losing a considerable amount of weight.

I mentioned it to the doctor and unfortunately, we can't remove it on the NHS and well there's no way that I can afford what they want to afford. So, you have to live with it. [Robert]

Attending to his language within this statement, his use of the pronouns *we* and *I* as reflecting a division between himself and the medical profession with regard to how best to manage his dissatisfaction with his body was understood. Developing the impact of this sense of division one step further, the assertion *you have to live with it* is understood as expressing a feeling of hopelessness around the possibility of obtaining the help he so desperately wants and of resigning himself to living with his dissatisfaction.

This subtheme highlighted the ways in which participants described feeling alone in managing their post‐operative bodies whether as a result of feeling that others are unwilling to help, with the aim of protecting others, through a fear of revealing their body with its signs of weight loss, or to avoid others judgement.

### 3.4 The Presence and Absence of Appropriate Support

Building on participants' experiences of their post‐operative bodies within their relational contexts, the final theme captures the many ways in which participants experienced support throughout their bariatric journeys. Although participants' accounts of support occur within relational encounters, such as family, partners, medical professionals and peers, their statements differ from those referred to within the previous subtheme in that statements relate to the ways in which support, and the absence of it, impacts upon their experience of adjustment following surgery and ability to renegotiate their sense of themselves, others and the world.

#### 3.4.1 The Absence of Appropriate Professional Support

Participants expressed the view that there exists a dearth of appropriate support from the medical profession following surgery. For some, this related directly to a lack of support in helping them manage new and unfamiliar physical symptoms resulting from the gastric by‐pass, whereas for others there was felt to be an absence of support in helping them adjust to the psychological impacts of the physical changes experienced following such rapid weight loss.

They could be a bit more explanatory and say what the side effects will be. Only recently has the surgeon said to me that well the three‐year honeymoon periods over it's now down to you to carry on losing weight. [Robert]

The dismay Robert expressed at the suggestion that he now needs to take responsibility for further weight loss highlights the misconception he entered his surgery journey with and how this has contributed to his sense of disillusionment.

#### 3.4.2 The Privileging of the Body in Post‐Operative Support

The second subtheme captures experience when in contact with medical professionals and how, within these encounters, they describe their psychological wellbeing as remaining unaddressed. Participants described experiencing the nature of encounters with bariatric support as impersonal, in which they regarded support as too generic or where their weight loss formed the focus of attention as opposed to their experience of post‐operative weight loss.

At the beginning it's made an issue about your psychological wellbeing but as soon as surgery's done, it's no longer an issue because it's never mentioned again. And maybe that's why you wouldn't bring it up because it's not there. It's not one of the tick boxes ‘how are you feeling emotionally?’ ‘How are relationships?’ It's not there. It's not a question that's asked. [Bronwyn]

#### 3.4.3 The Importance of Peer Support

In contrast to participants' accounts of feeling overlooked within professional services, there existed an acknowledgement of the ways in which support from peers had helped. An emphasis was placed on mutual understanding that only others who had undergone gastric bypass surgery could provide.

If you've never had the weight problem, you don't understand. So that's really good and it's really nice as well to be able to support them so you've got the two‐way thing. [Jenny]

Participants' experiences of support post‐operatively acknowledge a gap between the medical and the personal experience of weight loss and management of the physical body post‐operatively. Occupying the space between the two perspectives of post‐operative care, participants describe how they experience feelings of depersonalisation through the prioritisation and objectification of their physical body over their emotional well‐being by the medical profession, as well as the ways in which their experience of undergoing surgery forms a bond with peers who share the unique challenges seen as differentiating weight loss post‐surgery from that of non‐surgical weight loss.

## 4 Discussion

Overall, the purpose of this study was to gain an in‐depth understanding of the long‐term lived experience of those who have undergone RYGB surgery. In total, eight men and women took part in semi‐structured interviews which were analysed using IPA. Through a deep and sustained engagement with the participants accounts characterised by double hermeneutic engagement, four principal areas were identified within participants accounts of living with Roux‐en‐Y gastric bypass surgery: managing change and uncertainty; the affective experience of change; the post‐operative body within its relational context; and the presence and absence of appropriate support.

### 4.1 Change and Uncertainty Following Surgery

Participants noticed how their physiologies had changed following surgery and how they were now required to respond to these changes at cognitive, behavioural and emotional levels. Whilst each participant's experience of change differed markedly, they all described how they had to renegotiate their relationship with their body, either practically, or through the identification and attunement to affective sensations which they had not been aware of before. Rather than representing a linear process as suggested in some studies (e.g., Engström and Forsberg 2011; Ansari and Serjeant 2023), participants' experience of adjustment was characterised by ambivalence between new opportunities and feelings of loss and resentment towards the ways in which the imposed restriction from surgery meant they were unable to enjoy foods in the same way as before. A prominent feature of participants' experience of change was the observation that their bodies were no longer docile when it came to eating practices and instead required considerable thought and attention. This represents a pragmatic process of deduction or trial and error as to what their altered anatomy could tolerate, whereas for others the adjustments represented disruptions with habitual routines and their hectic schedules reminding them that ‘food is my life now’ [Bronwyn]. Studies acknowledging the experience of adjustment following bariatric surgery have referred to it as a phase (Ogden et al. 2006) and process (Warholm et al. 2014) noting the ways in which, through necessary changes to meal frequency and nutritional requirements food becomes a focus of participants' daily lives (Lier et al. 2015).

Whilst the findings from this study are in line with those acknowledging the process of adjustment following surgery, previous studies have looked at change over shorter periods of time. Whilst studies looking at the longer‐term have described the way in which eating can continue to be deeply problematic (Natvik et al. 2014), the current study suggests the process of ambivalence towards imposed restriction can be an ongoing experience for some in which food and eating continue to represent a major focus. In attempting to understand the ambivalence reported within the current study, Robertson's (2007) use of the term ‘edgework’ to describe risk‐taking behaviours as attempts ‘to transcend the banality of everyday existence’ (p. 49) offers a helpful perspective for understanding the diverse ways in which participants attempted to transcend the restriction imposed on them by the RYGB in attempts to reaffirm a sense of personal autonomy and social identity.

Although participants in the present study identified changes that they sometimes found difficult to negotiate, the sense of uncertainty experienced in the present was set against a belief in a certain future that would have awaited them had they not undergone bariatric surgery. This strategy would seem comparable to the strategy referred to by Engström and Forsberg (2011) where participants described reflecting on their previous condition of being obese as enabling them to both obtain and maintain control post‐operatively. Similarly, Alegria and Larson's (2014) participants spoke of the importance of purposely acknowledging improvements in their former self as a way of coping with the uncertainty brought about following surgery.

### 4.2 The Affective Experience of Change

Alongside the ways in which participants in this study acknowledged their experience of living *through* their bodies had been altered and now required attention, they also referred to ways in which their affective experience of living *in* their bodies had changed post‐operatively. Nearly all participants recognised feelings of disappointment at the disparity between what they hoped for following weight loss and the situation they now found themselves in. Some described now feeling ‘stuck’ or that parts of their body had not adjusted, suggesting that the changes that they had experienced had stopped short of what they had hoped for, whereas others acknowledged ways in which ongoing episodes of dumping restricted opportunities for self‐actualisation by preventing a return to work (Moss 1992).

Participants' disappointment with body image is consistent with previous studies that have highlighted the impact of loose skin experienced by many after undergoing bariatric surgery (Bocchieri et al. 2002; Groven et al. 2013; Warholm et al. 2014; Ockell et al. 2021). Indeed, whilst participants in the current study had lost considerable amounts of weight their experience of disappointment with their post‐operative bodies continued to incorporate the perspective of the Other, for example in Jenny's declaration that ‘I felt sexier when I was bigger […] just because it's normal […] fat is actually quite normal in our society […] Whereas saggy skin is not normal in our society.’. The impact of loose skin reported here might seem unsurprising when considered alongside research looking at individuals' expectations prior to surgery. For example, in a study by Homer et al. (2015), participants reported that despite being told of the risks of loose skin following surgery by the multi‐disciplinary team, they were unconcerned about the consequence of this happening to them and instead believed that achieving a smaller size would outweigh the potential negative impact from this.

In attempting to deal with the disappointment, participants alluded to a split between their body and self in attributing responsibility for the perceived failure, such as was the case in Sophie's description of how ‘my body didn't make it work’. Considering the possible function of this splitting, Moss (1982) described the way in which individuals can sometimes ‘present’ themselves in ways that attempt to avoid owning their visible appearance. Moss (1992) described how obese women described encountering their body as a thing and something from which they were estranged. Whilst Moss's view would seem consistent with the split between body and self which is observed in the current study, R. D. Laing's theory of ‘embodiment’ offers a more dynamic account of the intrapersonal experience in which the ‘individual experiences his self as being more of less divorced or detached from his body’ (1965, 69). Viewed in this way, the ‘detaching’ of oneself from the source of the disappointment, in this case the body, might offer a way of managing the tension between competing attributions individuals encounter when trying to understand their failure to lose weight.

An experience which further characterised participants' experience of living *in* their body was the ways in which they recognised, despite noticeable physical changes, continuing to perceive their bodies as large. Within their descriptions there once again appeared an underlying mind–body split that seemed to form a central part of their experience which was most articulately captured by Joyce describing having her ‘fat head’ on; a notion consistent with Souza et al.'s (2023) ‘ghost fat’ to describe how individuals who have previously been overweight continue to perceive themselves as such despite considerable weight loss. Given the influence of one's own sense of embodiment on their perception of body image, participants' uncertainty over their current physical state might act as an obstacle to assimilating their smaller bodies into their view of self.

### 4.3 The Body in Its Relational Context

Consistent with the phenomenological view of the body as ‘that which enables one to be seen by the Other’ (Moss 1992, 184) participants referred to ways in which encounters with others following surgery offered both opportunities to develop new views of themselves but also challenges to their previous views of self, others and the world. Explicit within these experiences were acknowledgments of how participants evaluated their bodies against social categories of normality, either in terms of size or in what they were able to participate in because of their smaller bodies. Despite describing ways in which they noticed their smaller bodies conforming to normative social markers, opening possibilities to redefine their sense of themselves as social beings, in other ways the body remained a source of ongoing distress which limited the extent to which they felt able to fully realise their smaller bodies, reinforcing a sense of isolation from others. Interestingly the experience of isolation experienced by participants comprised a quality of not being seen by others either by their own design, to avoid anticipated shame or concern, or through others not understanding the challenges involved in adjusting following bariatric surgery.

### 4.4 The Presence and Absence of Available Support

Explicit within the accounts was how support impacted upon the experience of adjusting following surgery and participants' ability to renegotiate their sense of themselves, others and the world. Participants described how they felt professional support failed to meet their individual needs. A theme that characterised participants' accounts of the perceived absence of support concerned the failure to address the emotional and psychological impacts of surgery. Participants described experiencing the procedures characterising the post‐operative follow‐up as representing a ‘box ticking’ process which appeared in distinct contrast to the preoperative screening they were required to go through. The qualitative shift between pre‐ and post‐operative support appeared to reinforce a body–mind split in which despite emotional wellbeing being ‘made an issue about’ prior to surgery the absence of any follow‐up regarding their emotional wellbeing was understood as indicating its lack of importance post‐operatively. Consequently, participants described feeling the support that was offered did not align with the challenges they encountered, but instead prioritised the physical aspects of weight loss through the practices of weighing and measuring as opposed to enquiring about their emotional wellbeing and relationships; a focus which some felt inhibited their willingness to raise the issues they were experiencing during consultations. Despite current guidelines emphasising the need for services to offer psychological support both pre‐ and post‐operatively (NICE 2016) there continues to be an absence of this, reflected both within the current study and elsewhere (MacAskill et al. 2023; Tolvanen et al. 2023; Faulkner and Lusher 2024b; Van Zyl et al. 2024).

### 4.5 Strengths and Limitations of the Current Study

A strength of this study lies in the focus on individuals who have undergone the RYGB procedure only. Most qualitative studies looking at post‐operative outcomes have tended to include samples who have undergone different surgical procedures (Chan et al. 2020; Youssef et al. 2021; Van Zyl et al. 2024). Research on individuals' reasons for and against different bariatric procedures has reported the perceived invasiveness, irreversibility and risks of complications associated with the RYGB as contributing to a person's decision to undergo alternative procedures (Opozda et al. 2017). Given the potential significance of these factors in a person's experience of post‐operative adjustment and the limitations they regard as being imposed on them, the current study's focus on one procedure reflects a strength through its focus on a surgically homogenous group.

Focusing on participants who had undergone surgery a minimum of 12 months previously also enabled an exploration of the longer‐term experiences of change post‐operatively. On average, participants in the current study had undergone surgery 24 months prior to taking part in the interviews, a point at which the initial restriction imposed by the bypass would have reduced and participants would have been reaching the end of the two‐year follow‐up recommended for post‐operative care (NICE 2016). Interviewing participants who were no longer experiencing the restriction imposed by the surgical procedure meant that they were able to reflect on the process of change and offered the possibility of considering what had been helpful throughout their post‐operative journey.

### 4.6 Future Directions

There is scope for future work to consider ways in which individuals can be supported throughout the post‐operative journey and the types of services that might be best suited to this. The current study was based on a sample of self‐selected participants from one bariatric centre in the South of England, UK, who remained in contact for, on average, 24 months. Relying on the process of self‐selection after receiving information during routine dietetic consultations meant that there was no way of knowing any information regarding those who decided against taking part. Future research might seek to recruit participants from various geographical locations. Whilst the issue of self‐selection is difficult to avoid, consideration might also be given to the recruitment of individuals who have stopped attending follow‐up care offered by the bariatric service. The role of peer support also featured prominently in this study, and whilst research has suggested involvement in weight loss support groups as positively associated with successful weight loss post‐operatively (Beck et al. 2012; Conceição et al. 2020), future research could explore the particular features of this resource and the possibility for it to form a more integral part of an individual's bariatric journey.

### 4.7 Implications for Nursing Practice

Pre‐surgery psychological assessment acts as an opportunity for individuals to familiarise themselves with a healthcare professional should they encounter difficulties adjusting or adhering to lifestyle and behavioural changes post‐surgery (Ratcliffe and Banting 2023). Despite this, psychological assessment represents a complex intersubjective encounter which is imbued with socially normative roles and expectations as well as the person's desires and hopes for normality (Homer et al. 2015). Our findings align in that pre‐operatively hopes for surgery outweigh concerns over post‐operative complications and a patient's suitability for surgery pre‐operatively might bear little relation to their post‐operative experience of change. It is therefore argued that the availability of post‐operative support addressing the individuals' experience of adjustment might be better placed. This would require a shift away from focusing on weight loss as a marker of success to a more holistic view of the person's overall wellbeing post‐operatively (Dandgey and Patten 2023). As nurses play a leading role in devising holistic treatment plans and communicating and coordinating care between healthcare specialists and patients, they are ideally placed to support the psychological wellbeing of bariatric surgery patients by ensuring a biopsychosocial approach to post‐operative care is implemented.

## 5 Conclusion

Four superordinate themes described participants' experiences of living with RYGB that provide insight into the deeply personal nature of changes and struggles experienced after undergoing gastric bypass surgery and the ways in which these are inextricably intertwined with the person's social, cultural and personal histories. The observed changes did not occur in a vacuum but were intertwined with social relationships, requiring renegotiation of how they understood others and themselves within social encounters. This research offers insight into the experiences of individuals following Roux‐en‐Y gastric bypass surgery that can be used in nursing practice to inform the development of appropriate ways in which to holistically support individuals who made the decision to undergo bariatric surgery.

## Author Contributions

N.F. and A.V., Made substantial contributions to conception and design, or acquisition of data or analysis and interpretation of data. N.F., J.L. and A.V., Involved in drafting the manuscript or revising it critically for important intellectual content. N.F., J.L. and A.V., Given final approval of the version to be published. Each author should have participated sufficiently in the work to take public responsibility for appropriate portions of the content. N.F., Agreed to be accountable for all aspects of the work in ensuring that questions related to the accuracy or integrity of any part of the work are appropriately investigated and resolved.

## Conflicts of Interest

The authors declare no conflicts of interest.

## Peer Review

The peer review history for this article is available at https://www.webofscience.com/api/gateway/wos/peer‐review/10.1111/jan.16890.

## Data Availability

The research data is not shared beyond the data included in the article.
